# Effect of Pore Structure Heterogeneity of Sandstone Reservoirs on Porosity–Permeability Variation by Using Single–Multi-Fractal Models

**DOI:** 10.1021/acsomega.3c09957

**Published:** 2024-05-23

**Authors:** Peng Yao, Junjian Zhang, Zhenyuan Qin, Aiping Fan, Guangjun Feng, Veerle Vandeginste, Pengfei Zhang, Xiaoyang Zhang

**Affiliations:** † College of Earth Sciences & Engineering, Shandong University of Science and Technology, Qingdao 266590, China; ‡ School of Resources and Earth Science, China University of Mining and Technology, Xuzhou 221116, China; § Department of Materials Engineering, KU Leuven, Campus Bruges, Bruges 8200, Belgium; ∥ Department of Mechanical, Materials and Manufacturing Engineering, Faculty of Engineering, University of Nottingham, Nottingham NG7 2RD, United Kingdom

## Abstract

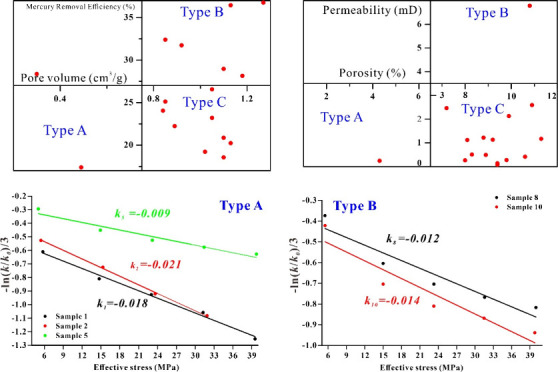

Pore structure heterogeneity affects sandstone porosity and permeability and thus sandstone gas productivity. A total of 17 sandstone samples collected from the northwestern margin of the Junggar Basin in Xinjiang Province are investigated in this study. The pore-fracture system distribution of target sandstones is studied by high-pressure mercury injection tests. On this basis, single- and multi-fractal models are used to characterize pore structure heterogeneity, and the applicability of four models (*Menger* model, *Sierpinski* model, *Thermodynamic* model, multifractal model) to characterize pore and fracture distribution heterogeneity are discussed. Moreover, a correlation between fractal dimension, pore structure parameters, and variation coefficient of porosity–permeability is discussed based on overburden permeability test results. The results are as follows. (1) *D*_S_ (fractal dimension of *Sierpinski* model) shows a significant correlation with pore volume percentage, so the Sierpinski model could better characterize fracture distribution heterogeneity quantitatively. Multifractal dimensions are consistent with those of Sierpinski and Thermodynamic models, which indicates that the single- and multiple-fractal models are consistent. (2) The porosity and permeability decrease as a power function with higher confining pressure. The porosity and permeability behavior changes at a critical conversion pressure value. For a confining pressure lower than this critical value, the porosity and permeability decrease largely. For confining pressures higher than this critical value, the porosity and permeability vary less. In contrast, permeability has a larger variation rate and is more obviously affected by confining pressure. (3) Pore compression space is affected by the permeability variation coefficient. Compressibility, porosity, and permeability variation coefficient have no relationship with pore structure parameters since compressibility is affected by pore structure, mineral composition, and other factors in sandstone samples.

## 1 Introduction

China is rich in natural gas resources, accounting for about 10% of global resources. However, tight sandstone gas production capacity is limited, since these natural gas reservoirs are characterized by low porosity and permeability.^1−3^ Relevant studies indicated that the pore structure of sandstone reservoirs has an important influence on fluid migration. Strong heterogeneity in pore structures impedes migration of fluids or gas and thus leads to a reduction in natural gas production and recovery efficiency.^4−8^ Therefore, the pore structure of tight sandstone reservoirs is an important factor that contributes in determining reservoir physical properties and oil/gas productivity.^9^

The pore structure of sandstone reservoirs was thus studied here, using low-temperature liquid nitrogen tests (LPN_2_ GA), carbon dioxide adsorption tests (LPCO_2_ GA), low-field nuclear magnetic resonance technology (LF-NMR), overburden permeability tests (DP-P), high-pressure mercury injection tests (HPMI), and scanning electron microscopy (SEM).^10−16^ Among those techniques, HPMI has been the most common method to study sandstone pore structure parameters due to its advantages of speed, convenience, and larger pore range.^17^ Lai et al.^18^ indicated that pore geometry, pore distribution, and pore connectivity are important for improving oil recovery. Wang et al.^19^ showed that frequent alternation of different diameter pore sizes and increased fluid flow resistance is related to the poor seepage capacity of sandstone gas reservoirs. Miao et al.^20^ suggested that the pore and fracture distribution heterogeneity (PFDH) is negatively correlated with interstitial materials. In conclusion, the fractal dimension of sandstone as a whole is larger, pore distribution is uneven, and pore shape is complex.^21−23^

Several studies applied fractal theory to characterize the pore structure quantitatively.^24,25^ Currently, the fractal models include the single fractal model *Menger* model (M model), *Sierpinski* model (S model), *Thermodynamic* model (T model), etc.), and multifractal model.^26,27^ Su et al.^28^ showed that single fractal dimensions *D*_1_ and *D*_2_ could represent the complexity of pore structures. Hao et al.^29^ showed a negative linear correlation between fractal dimension and porosity/permeability. However, different from the single fractal model, the multifractal model could be more accurate for the study of PFDH.^30−33^ Relevant research has proved that pore fractal features of tight sandstone have multiple fractal structures for different pore diameters.^34−37^

Dynamic changes in porosity and permeability restricted by pore structure have been investigated in several works.^38−42^ Sun et al.^43^ suggested that the fractal dimension is negatively correlated with porosity and permeability. Li et al.^44^ found that the permeability in sandstone is negatively correlated with stress. In the compaction stage, the porosity decreases. At the beginning of the elastic stage, porosity is negatively correlated with stress after the porosity increases. In the unstable fracture stage, the porosity also increases.

In conclusion, dynamic variations of porosity and permeability restricted by pore structure have been studied. However, there are still several knowledge gaps in this research field. First, further research is needed on the dynamic variations in permeability linked to pore-fracture structure. Second, the applicability of single–multi-fractal models to characterize PFDH in sandstone reservoirs needs to be further explored and the PFDH factors that control gas productivity need to be determined.

Therefore, a total of 17 samples from the Mahu Sag at the northwestern margin of the Junggar Basin have been selected for this study. The pore distribution of the target sandstone is characterized using SEM and HPMI. The samples are divided into different types according to the mercury intrusion pore parameter results. Then, single- and multi-fractal models are used to characterize the PFDH quantitatively and the applicability of the classification model to characterize PFDH is discussed. Moreover, the correlation between the fractal dimension, pore structure parameters, and variation coefficient of pore permeability is evaluated using DP-P. It should be noted that this article adopts the Hodot reservoir pore classification scheme, which divides the pores into micropores (<100 nm), meso-pores (100–1000 nm), and macro-pores (>1000 nm).^45^

## 2 Study Area and Experimental Methods

### 2.1 Sample Preparation

**Figure 1:**
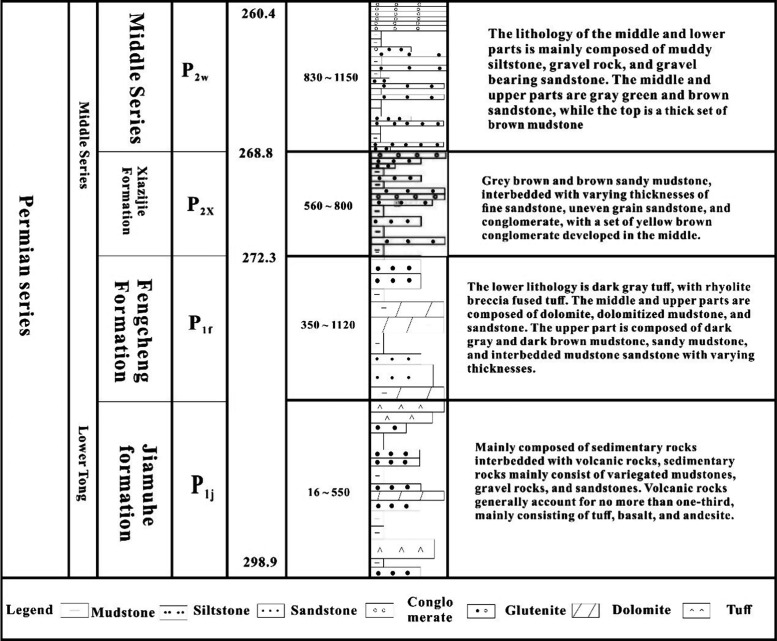
Sampling location and lithology bar chart.

Mahu Sag is located northwest of the central depression of the primary structural unit in the Junggar Basin, which is one of the secondary structural units with the largest oil and gas reserves in the basin and is also recognized as the most hydrocarbon-rich sag. The depression has an area of approximately 4260 km and is generally distributed in a northeast–southwest direction. The longest in the northeast direction is about 120 km, and the widest in the southwest direction is about 35 km. In the Late Carboniferous, the collision between the Junggar Block and the Tacheng Block caused the western Junggar orogenic belt to be thrust into the basin, and the Mahu Depression began to take shape. In the early Permian, the Junggar Block and the Kazakhstan Plate underwent intense collision and compression, leading to further subsidence of the Western Junggar Ocean and the gradual formation of the foreland-type Mahu Depression. During the Late Permian to Triassic, the tectonic reversal caused the formation of the Wuxia and Kebai fault zones in the western Junggar region, leading to tectonic uplift in the western part of the depression (Figure 1).^46^

The study area is located in the Mahu Sag (an area of about 5000 km^2^) at the northwestern side of the central depression of the Junggar Basin (China). It lies next to the Xiayan uplift and Dabasong uplift areas in the southeast, the Yingxi depression and quartz beach depression areas in the northeast, the eastern margin of the Zhongguai uplift area and Kebai fault zone in the west, and the Wuxia fault zone in the north. The structure of the Mahu sag is relatively simple, with a southeast tilt monocline and locally small amplitude structures.^47,48^ A continental sedimentary sequence of more than 8000 m, with mainly the Permian Fengcheng Formation, is superimposed on the Mahu sag. In total, 17 sandstone samples are collected from Well XX in the Mahu sag area, and the basic data of all samples are shown in Table 1.

**Table 1:** Basic Information of Sandstone Samples

sample no.	total pore volume (cm^3^·g^–1^)	porosity (%)	permeability (mD)	mercury removal efficiency (%)
1	1.10	9.4	0.077	20.87
2	0.85	7.2	2.46	25.12
3	1.27	11.3	1.17	36.75
4	1.18	10.8	6.78	28.15
5	1.10	9.2	1.13	28.97
6	1.13	9.8	0.276	36.45
7	0.85	8.1	1.12	32.39
8	0.89	8.8	1.22	22.23
9	0.92	8.3	0.504	31.74
10	1.05	8.9	0.492	23.21
11	1.10	10.9	2.59	18.55
12	1.13	9.9	2.13	20.23
13	0.49	4.3	0.242	17.38
14	0.84	8.0	0.266	24.05
15	1.02	9.4	0.141	19.22
16	1.05	10.6	0.415	26.56
17	0.30	2.5	<0.010	28.36

### 2.2 Experimental Methods

**Figure 2:**
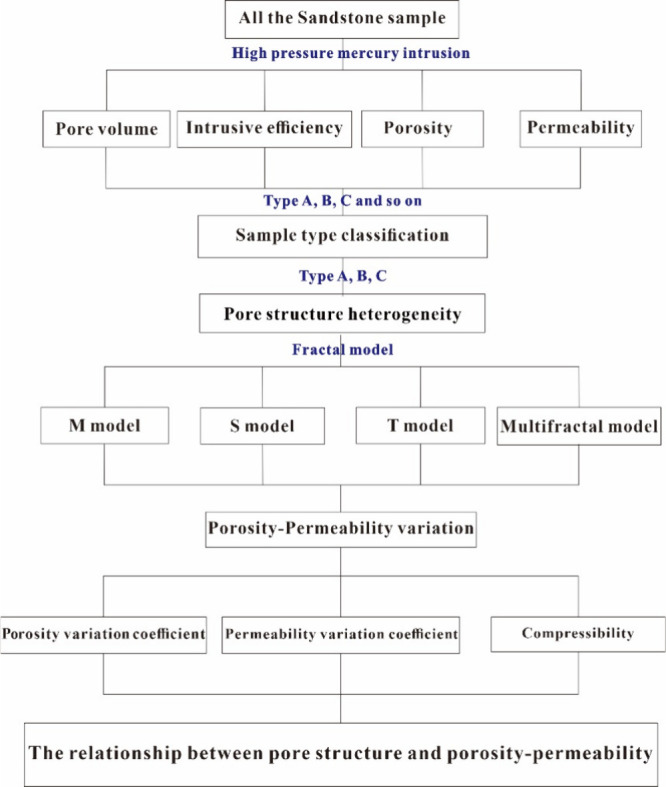
Principle and experimental methods of all of the samples.

Figure 2 shows the theoretical analysis and experimental method flowchart. The specific experiment is described in detail below.

#### SEM Tests

Small square parts of the samples are cut, impurities on the sample surface are removed with nitrogen, and the surface is bombarded with an argon ion beam to obtain a polished flat surface. After completion of the sample preparation, a Quanta 250 scanning electron microscope is used for testing and SEM photos are quantitatively analyzed with PCAS software. The surface morphology of the samples is then identified by the high-resolution SEM photos.

#### HPMI Tests

Before the experiment, samples are dried at 105 °C until constant weight. A 9500 mercury intrusion meter is used to conduct pore-fracture experiments. The maximum experimental pressure is 30 MPa, the aperture measurement range is 0.05–2.5 μm, and the test temperature is 25 °C. The specific surface area and pore distribution are obtained by varying the pressure.^49^

#### DP-P Tests

Before the experiment, the porosity and permeability of the samples under initial conditions are measured and then the core is put into a YC-4-type overburden pore permeability tester to start the experiment. The displacement pressure is kept unchanged, and the overburden pressure is gradually increased by controlling the confining pressure. The confining pressure is set within the range of 5–40 MPa. Similarly, the confining pressure is controlled to gradually reduce the overburden. Once the overburden point pressure stabilizes, the porosity and permeability under different effective overburden pressures are measured. Finally, the data are analyzed and processed.^50^

### 2.3 Calculation Theories

The *Menger* model is shown in eq 1:^30^
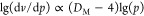
1where *D*_M_ is the fractal dimension of the *Menger* model, dimensionless; *P* is the injection pressure, MPa; and *V* is the total injection volume, cm^3^·g^–1^.

The *Sierpinski* model is shown in eq 2:^49^
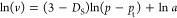
2where *D*_S_ is the fractal dimension of the *Sierpinski* model; *P*_t_ is the threshold pressure, MPa; and *a* is a constant.

The *Thermodynamic* model is shown in eq 3:^31^
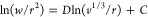
3where *r* is the pore radius, nm, and *D* is the slope of eq 3 and represents the fractal dimension of the *Thermodynamic* model.

*q ∼ D*(*q*) describes the local features of the multifractal. The calculation formula of *D*(*q*) is as follows:^27^
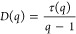
4where τ(*q*) is the mass exponential function and *q* is the statistical moment order.

When *q* = 1, the calculation formula for *D*(*q*) becomes
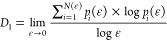
5where ε is the scale; *P*_*i*_ is the probability value of nitrogen adsorption volume in the *i*th interval.

The relationship between generalized fractal dimension and multifractal spectrum conforms to the *Legendre* transform, and its expression is
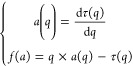
6

The equation constructs independent variables *q* and τ. The relationship between independent variables *a* and *f* can both be used to describe multifractal features, where *f*(*a*) is the fractal dimension of subsets with the same singularity index; *q* is the statistical moment order; and *a* is the set of singularity indices.

The DP-P test could be used to obtain *C*_f_.
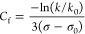
7where *C*_f_ is the pore compression coefficient under the variation of horizontal effective stress, MPa^–1^; *k* is the permeability after the horizontal stress changes to σ MPa, mD; and *k*_0_ is the initial permeability, mD.

The permeability variation rate is introduced to describe permeability sensitivity, shown as^42^
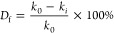
8where *D*_f_ is the permeability variation rate; *k*_0_ is the initial permeability, mD; and *k*_*i*_ is the permeability under confining pressure, mD.

The dimensionless parameter, porosity variation rate, is introduced to describe permeability sensitivity, shown as^45^
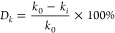
9where *D*_*k*_ is the rate of porosity change; *k*_0_ is the initial porosity, mD; and *k*_*i*_ is the porosity under confining pressure, mD.

## 3 Results and Discussion

### 3.1 Experimental Results of Scanning Electron Microscopy

**Figure 3:**
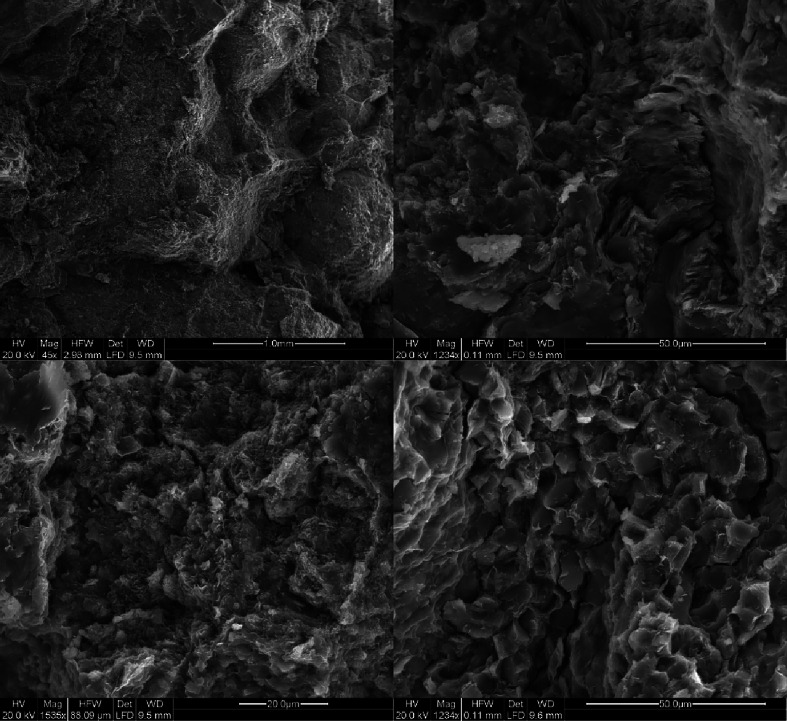
Scanning electron microscopy sample image.

Through scanning electron microscopy analysis, the tight sandstone reservoir exhibits rich and complex nano-scale pore characteristics, mainly including intergranular pores of clay minerals, residual intergranular pores, dissolution pores within particles, and microcracks. Clay minerals fill the pores between particles, forming many intergranular pores of clay minerals, mainly composed of sheet-like chlorite, illite/montmorillonite-mixed layers, and kaolinite, with intergranular filling of albite, quartz, and carbonaceous minerals. The sample pores are undeveloped, and the connectivity is poor. Chlorite exhibits a regular structure in morphology but mostly presents a disorderly state of accumulation. Its intergranular pores include various forms such as cylindrical, slit-shaped, strip-shaped, and layered, with pore diameters ranging from 150 to 350 nm, averaging about 240 nm. The overall distribution of the Yimeng mixed layer is network-like, and its intergranular pores are mainly cylindrical, with pore diameters ranging from 280 to 990 nm, averaging about 630 nm. The physical properties of nanopores are poor, with a low oil content, and intergranular pores mainly exist in crude oil in the form of oil films. In tight reservoirs, the development of microcracks significantly improves the permeability of the reservoir, although the oil content of nanopores is relatively poor (Figure 3).

### 3.2 Sample Type Division Based on Mercury Injection Results

**Figure 4:**
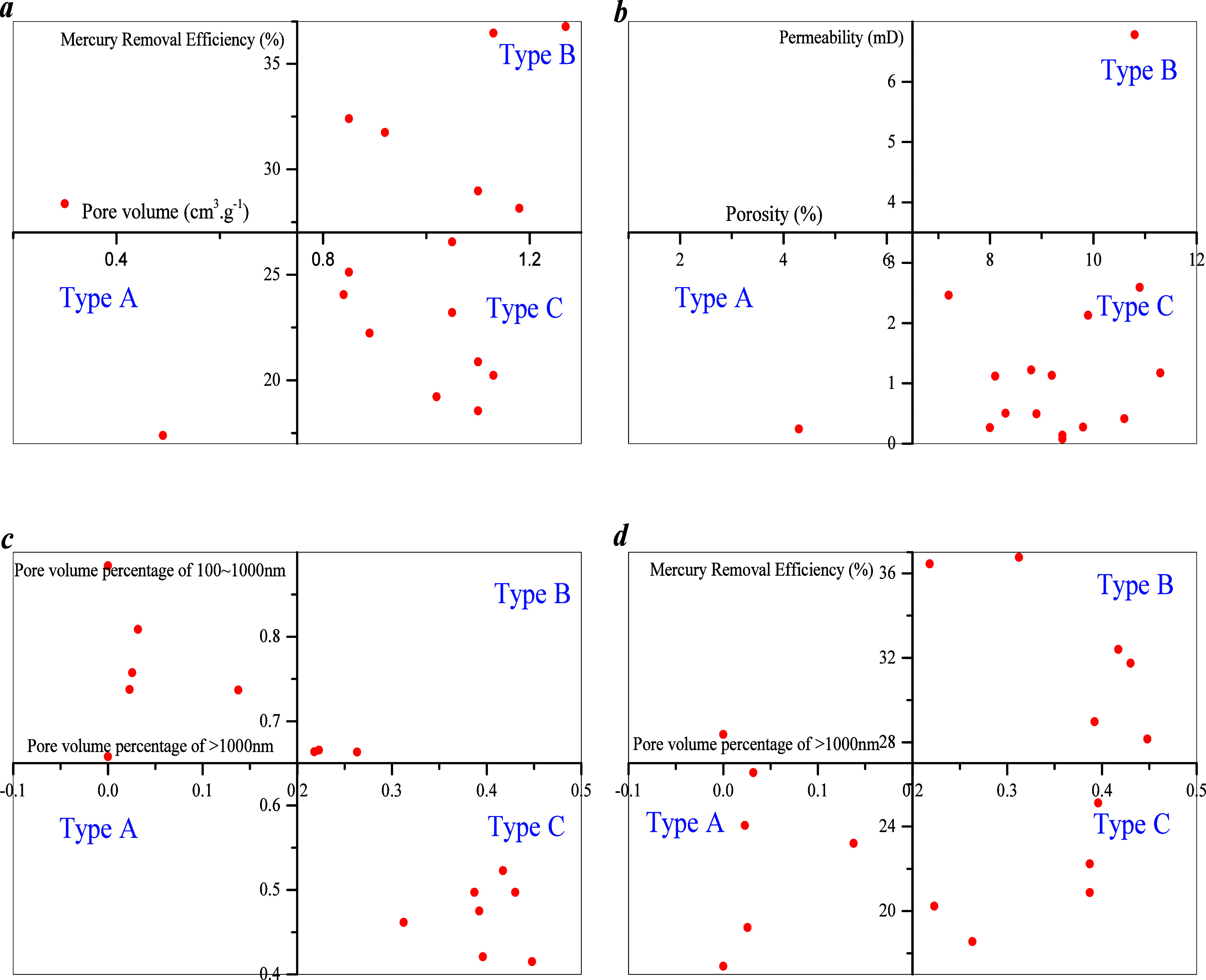
Sample division based on unconditional conditions: (a) pore volume and mercury removal efficiency; (b) porosity and permeability; (c) pore volume percentage >1000 nm and pore volume percentage of 100–1000 nm; (d) pore volume percentage >1000 nm and mercury removal efficiency.

Considering the sample data presented in Table 1 and the HPMI results, all samples are analyzed based on the correlations between pore volume–mercury removal efficiency, porosity–permeability, pore volume percentage (macro-pore)–pore volume percentage (meso-pore), and pore volume percentage (macro-pore)–mercury removal efficiency (Figure 4). The results show that the pore volume percentage–mercury removal efficiency correlation can be used as a basis for classification of the samples. Since PFDH affects recovery efficiency, pore development is reflected by pore volume. All samples are divided into three types based on pore volume percentage and mercury–removal efficiency. Type A involves developed micropores, a pore volume percentage of macro-pores of less than 20%, and mercury removal efficiency of less than 27%. Types B and C involve developed macro-pores, with a pore volume percentage of macro-pores that is greater than 20%. The mercury removal efficiency of type B samples is greater than 27%, and that of type C is less than 27%.

**Figure 5:**
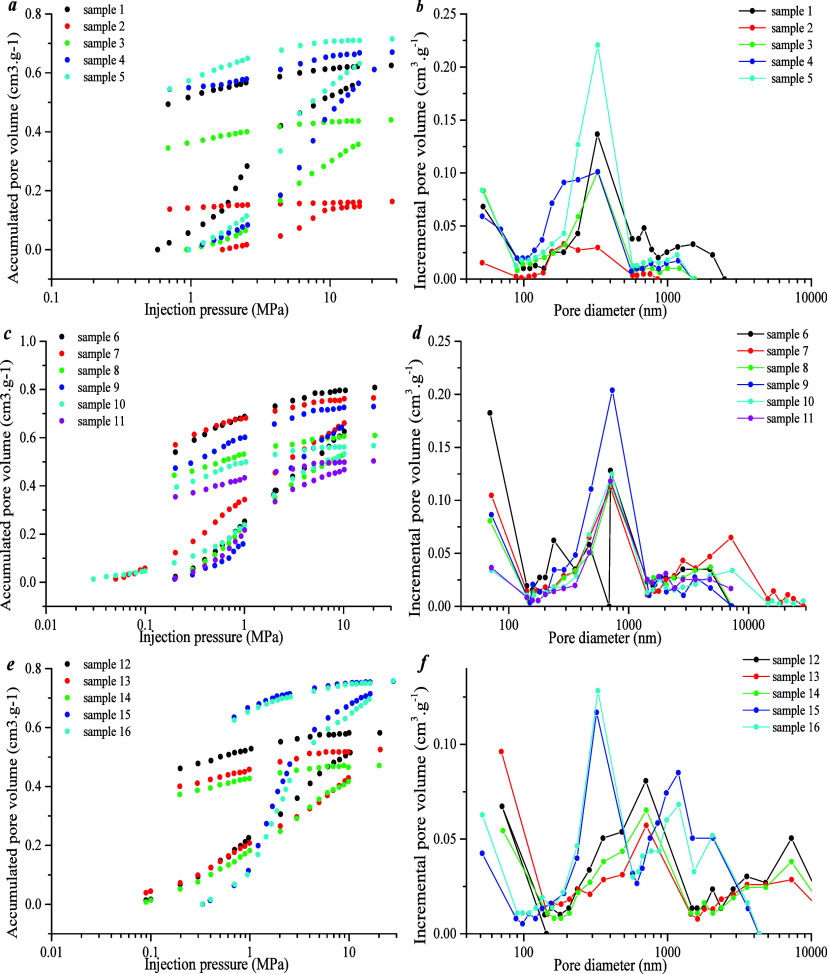
(a, c, e) Mercury intrusion and removal curve. (b, d, f) Stage mercury intrusion under different pore diameters.

The mercury injection results show that the mercury inlet pressure mainly falls in the range of 0.6–30 MPa. The mercury removal efficiency of type B is highest (35 to 55%). Mercury removal efficiencies of types A and C are similar (12 to 33%) (Figure 5a, c, and e). The pore diameter of type A mainly varies between 200 and 600 nm, and the pore volume percentage is 30–51%. The pore diameter of type B ranges between 360 and 1500 nm, and the pore volume percentage is 25–51%. However, the pore diameter of type C is evenly distributed, and the pore-throat diameter varies between 100 and 1000 nm (Figure 5b, d, and f).

**Figure 6:**
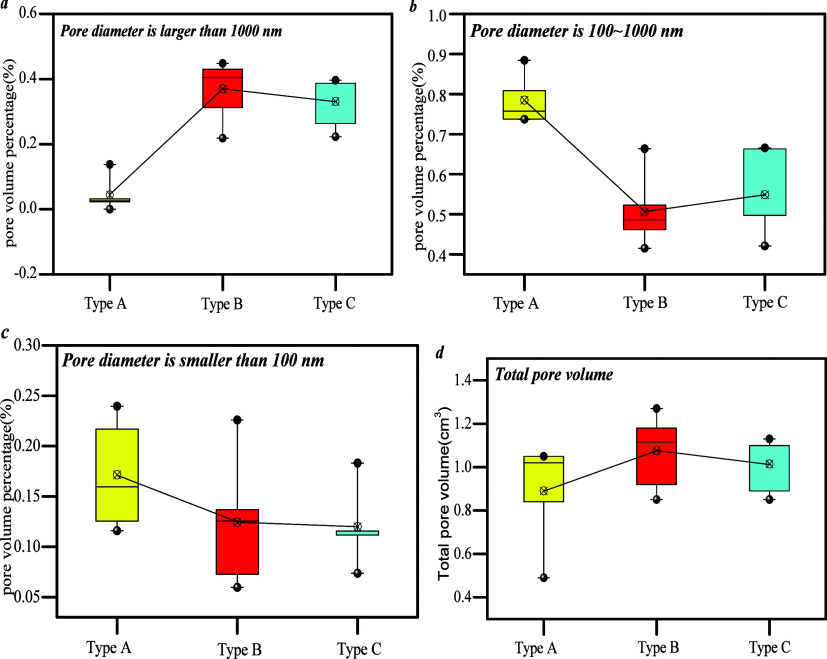
Comparison of incremental pore volume in different type samples: (a) pore diameter is larger than 1000 nm; (b) pore diameter is 100–1000 nm; (c) pore diameter is smaller than 100 nm; (d) total pore volume.

Figure 6a shows that the macro-pore volume percentage of type B ranges from 22 to 45%, that of type C from 22 to 40%, and that of type A from 2 to 14%. The meso- and macro-pore volume percentages of types B and C are higher than those of type A. Figure 6b shows that the percentage of meso-pore volume in type A is larger than that of types B and C. The meso-pore volume percentage of type A varies from 74 to 88%, that of type B from 42 to 66%, and that of type C from 42 to 67%. Figure 6c shows that the micropore volume percentage in type A is between 12 and 22%, that of type B between 7 and 14%, and that of type C mainly between 11% and 12%. Therefore, the micropore volume percentage in type A is higher than that of types B and C. In conclusion, micro-pores and meso-pores of type A are developed, and macro-pores of types B and C are relatively developed (Figure 6d).

### 3.3 Pore Size Distribution Heterogeneity by Using Fractal Dimension

**Figure 7:**
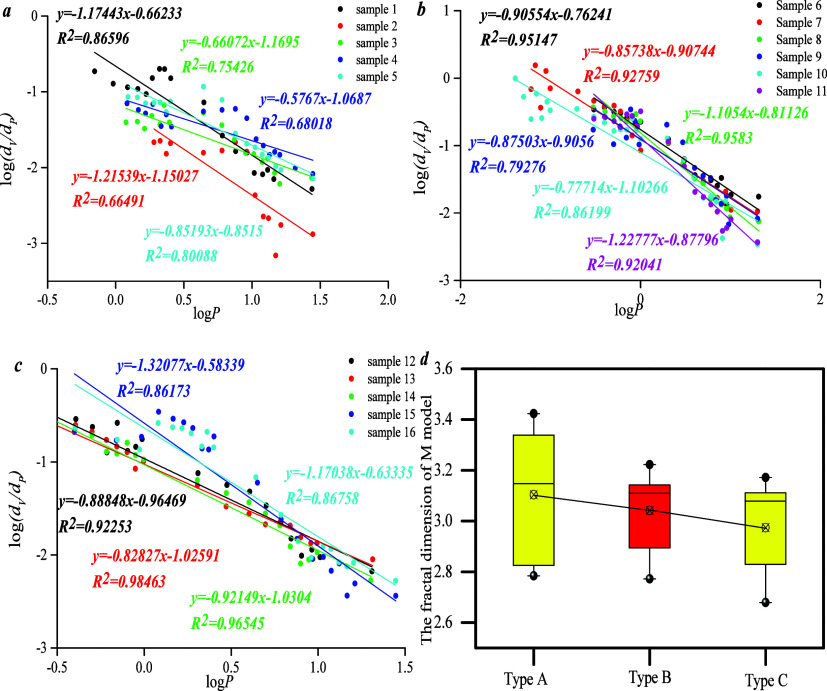
Fractal curves of all the samples based on model *M*: (a) the fractal curve of type A; (b) the fractal curve of type B; (c) the fractal curve of type C; (d) the fractal dimension of three types.

*D*_M_ could be calculated by the M model. The results show that the fractal curve could reflect a negative linear correlation between log *p* and log(d*v*/d*p*), indicating that the fractal dimension could be represented by this model. Differing from types B and C, *D*_M_ of type A is highest, indicating that the PFDH of type A is stronger than that of types B and C (Figure 7).

**Figure 8:**
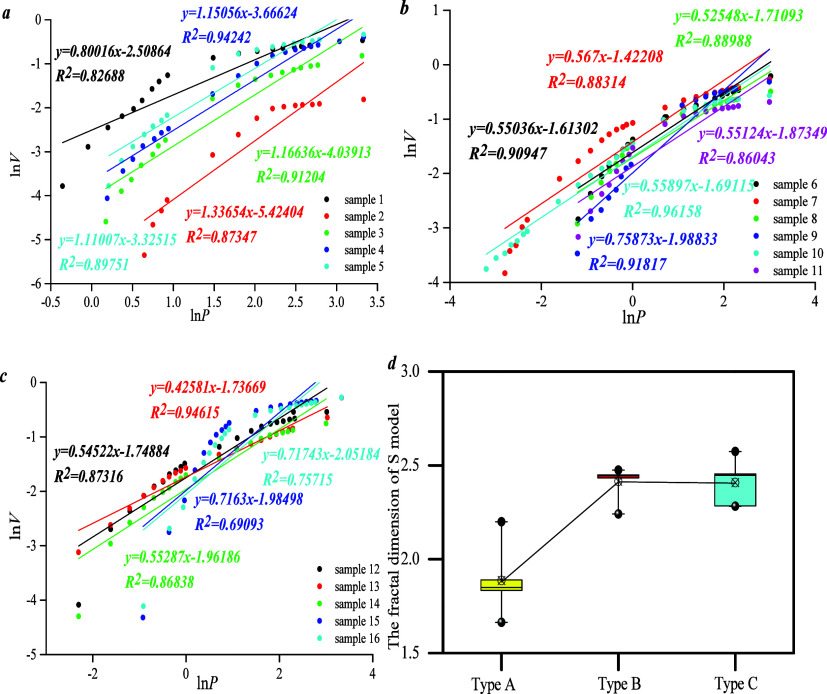
Fractal dimension of different type samples by using model S: (a) the fractal curve of type A; (b) the fractal curve of type B; (c) the fractal curve of type C; (d) the fractal dimension of three types.

*D*_S_ could be calculated by the S model. Results show that the fractal curve could reflect a positive linear correlation between ln *p* and ln *v*, indicating that the fractal dimension could be reflected by this model. *D*_S_ of type C overlaps with that of type B and is higher than that of type A, indicating that PFDH of types B and C is stronger than that of type A (Figure 8). Results of this model are inconsistent with the M model because the M model represents the complexity of the rock surface area whereas the S model represents the roughness of the rock pore volume.

**Figure 9:**
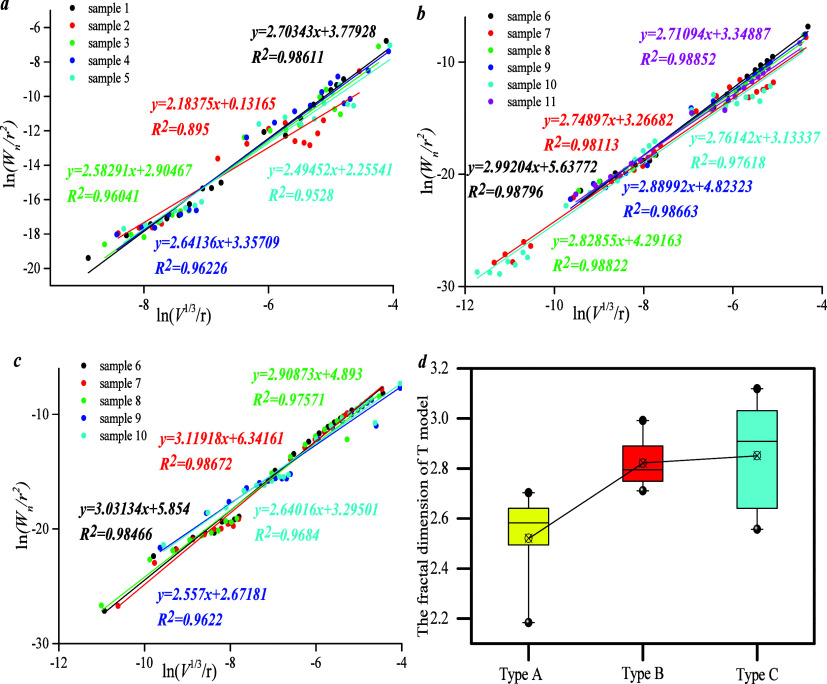
Fractal dimension of different type samples by using model *T*: (a) The fractal curve of type A; (b) the fractal curve of type B; (c) the fractal curve of type C; (d) the fractal dimension of three types.

*D*_T_ could be calculated by the T model. Results show that the fractal curve reflects a positive linear relationship between ln(*V*^1/3^/*r*) and ln(*W*/*r*^2^), indicating that the fractal dimension could be better reflected by this model. The linear fitting degree of type A varies from 0.9 to 0.99, and *D*_T_ ranges from 2.2 to 2.7. The linear fitting degree of type B ranges from 0.98 to 0.99, and *D*_T_ from 2.7 to 3.0. The linear fitting degree of type C falls between 0.96 and 0.99, and *D*_T_ falls between 2.6 and 3.1 (Figure 9). Thus, *D*_T_ of type C overlaps with that of type B and is higher than that of type A, indicating that PFDH of types B and C is stronger than that of type A. Results of this model are consistent with the S model, indicating that the T model also represents the roughness of the rock pore volume.

**Figure 10:**
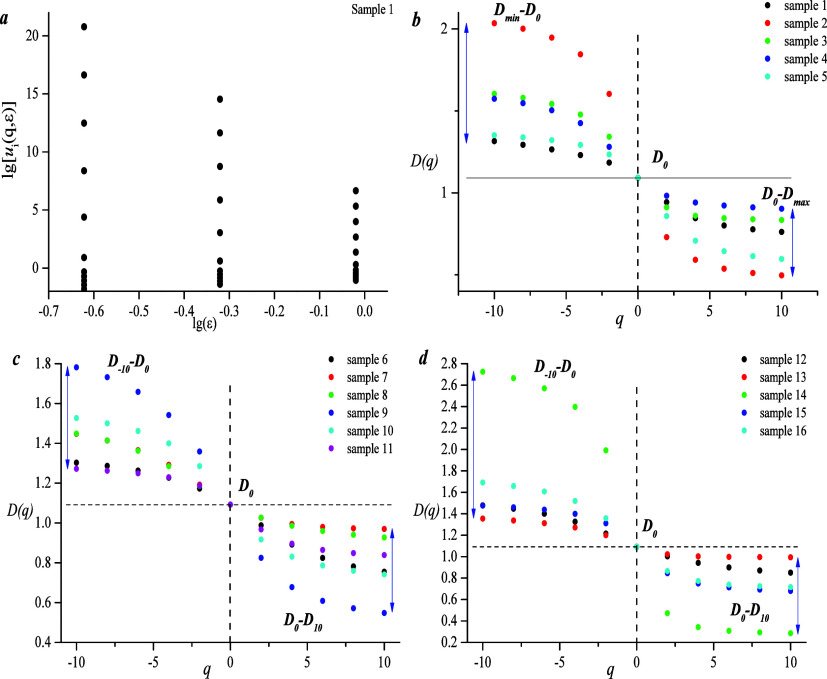
(a) lg(*e*)∼ lg[*ui*(*q*,*e*)] double logarithmic graph. (b–d) Multifractal curve of the A, B, and C types.

According to the generalized fractal dimension spectrum, *q* ∼ *D*(*q*) spectra of all samples show an obvious inverse S type, indicating that pore diameter distribution has characteristics of multifractal and the pore structure is clearly heterogeneous. *D*_–10_–*D*_0_ of type A is 1.2–2, and *D*_0_–*D*_10_ is 0.5–0.98. *D*_–10_–*D*_0_ of type B is 1.2–1.8, and *D*_0_–*D*_10_ is 0.5–1. *D*_–10_–*D*_0_ of type C is 1.2–2.7, and *D*_0_–*D*_10_ is 0.3–1. Relevant literature shows that the spectral width on the left represents the lower value pore volume region and the spectral width on the right represents the high-value pore volume region. Therefore, these results indicate that micropore heterogeneity of type C is strong, whereas the macro-pore distribution of type A is uniform (Figure 10).

**Figure 11:**
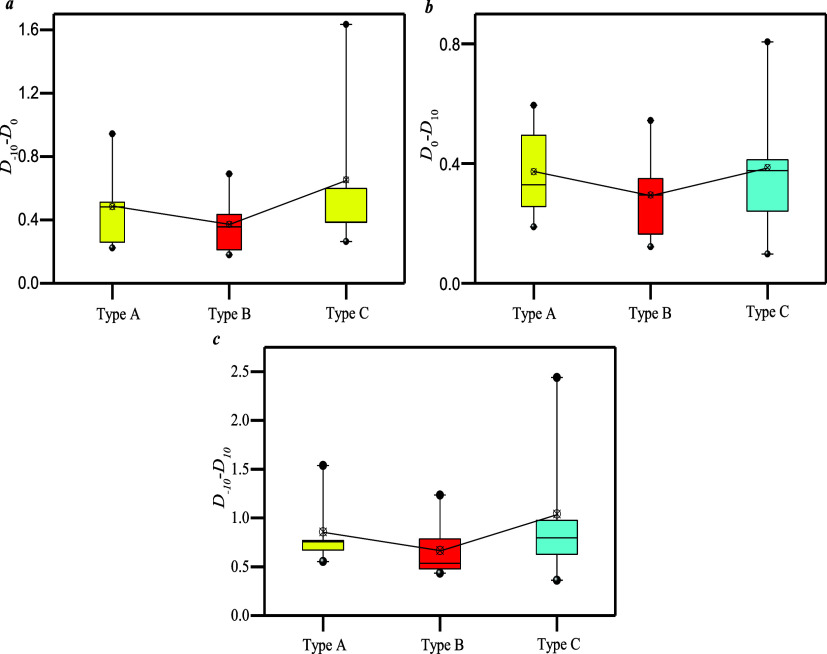
(a) *D*_–10_–*D*_0_ of three types; (b) *D*_0_–*D*_10_ of three types; (c) *D*_–10_–*D*_10_ of three types.

Figure 11a shows that *D*_–10_–*D*_0_ of type C is 0.26–1.63, which is larger than that of types A and B, indicating that type C has strong heterogeneity of micropores, which is consistent with results of Figure 10. Figure 11b shows that *D*_–10_–*D*_0_ of type A is 0.19–0.59, which is larger than that of types B and C, indicating that macro-pore heterogeneity of type A is significant, which is also consistent with results of Figure 10.

**Figure 12:**
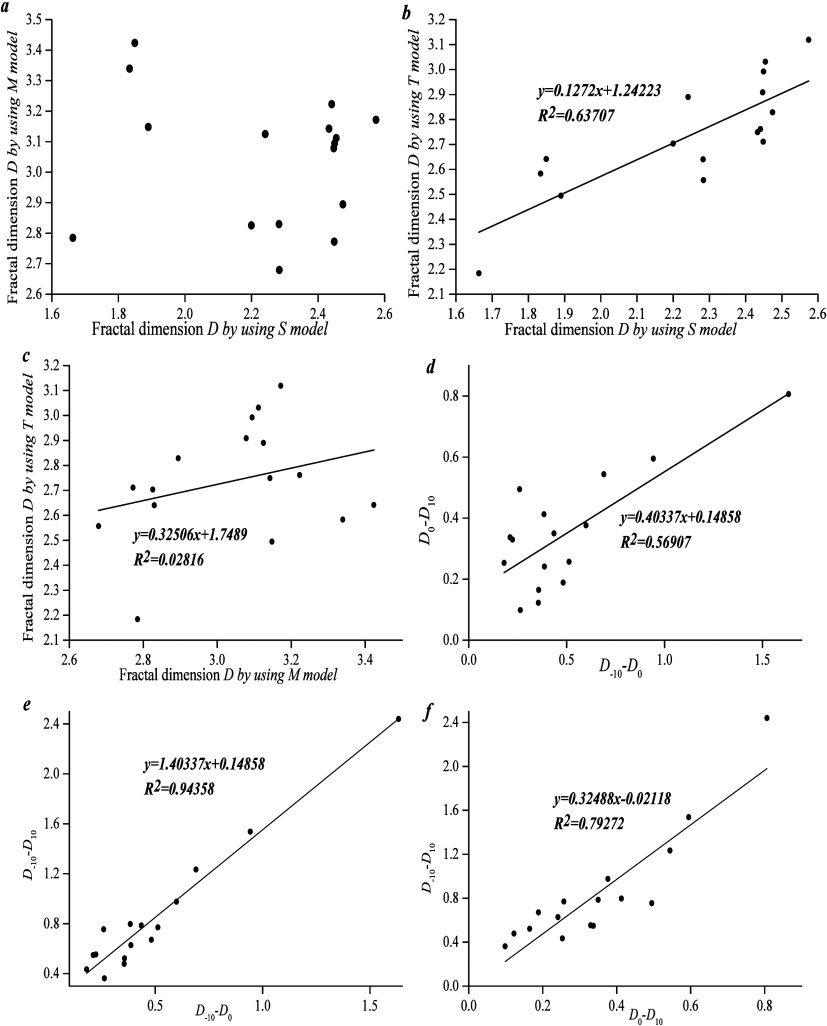
(a–c) Relationship between three single fractal dimensions. (d–f) Relationship between three types of multifractal dimensions.

Figure 12 shows that the correlation between *D*_S_ and *D*_M_ and between *D*_M_ and *D*_T_ is weak whereas *D*_S_ and *D*_T_ show a positive linear correlation, which indicates that the S model is more suitable for the characterization of reservoir pore structures. In addition, the multifractal calculation shows that *D*_–10_–*D*_0_ and *D*_0_–*D*_10_, *D*_–10_–*D*_0_ and *D*_–10_–*D*_10_, and *D*_0_–*D*_10_ and *D*_–10_–*D*_10_ show a positive linear correlation and the correlation between *D*_–10_–*D*_0_ and *D*_–10_–*D*_10_ is more pronounced. It is shown that the PFDH is affected by the lower pore volume region, which is determined by the pore distribution.

**Figure 13:**
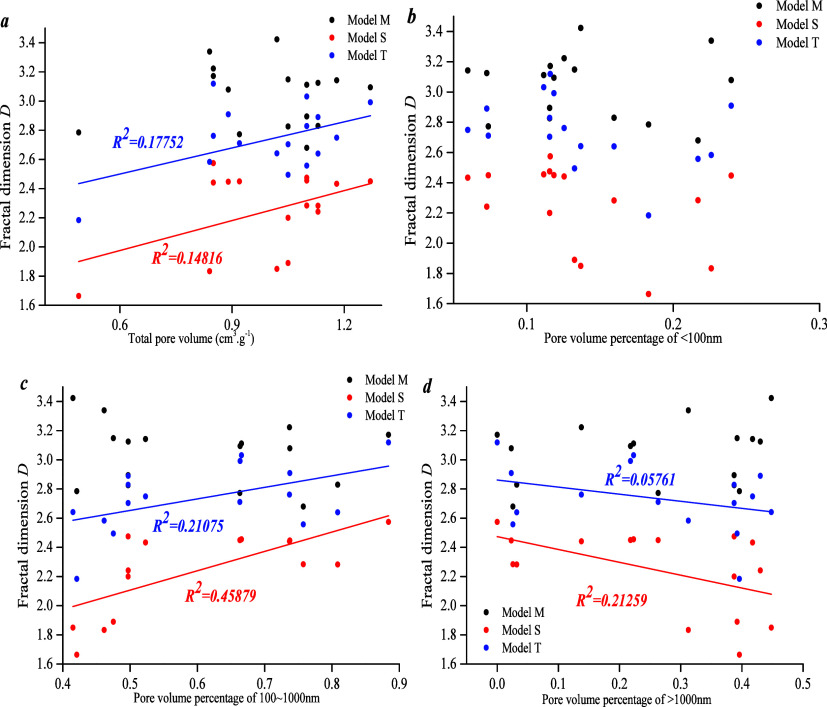
Correlation between pore volume and single fractal dimension: (a) total pore volume; (b) pore volume percentage of <100 nm; (c) pore volume percentage of 100–1000 nm; (d) pore volume percentage of >1000 nm.

Figure 13a shows that the total pore volume does not show a clear correlation with *D*_M_, but it does show a positive linear correlation with *D*_S_ and *D*_T_, indicating that PFDH is not reflected by the M model. Figure 13b shows that there is no correlation between micropore volume percentage and *D*_M_, *D*_S_*,* and *D*_T_, indicating that PFDH cannot be reflected by micropore volume percentage. Figure 13c shows that the meso-pore volume percentage does not have a clear correlation with *D*_M_, but that a weak positive linear correlation could be found with *D*_S_ and *D*_T_, and that the correlation with *D*_S_ is more significant, indicating that meso-pore distribution heterogeneity could be characterized by the *D*_S_ model.

Figure 13d shows that the macro-pore volume percentage has a weak negative correlation with *D*_S_ and *D*_T_, and no correlation with *D*_M_, indicating that macro-pore distribution heterogeneity could be characterized by the *D*_S_ model. In summary, *D*_S_ shows a weak correlation with pore volume percentage at different stages, and thus, the S model could be used for quantitative characterization of PFDH.

### 3.4 Effect of Confining Pressure on Porosity–Permeability Variation

**Figure 14:**
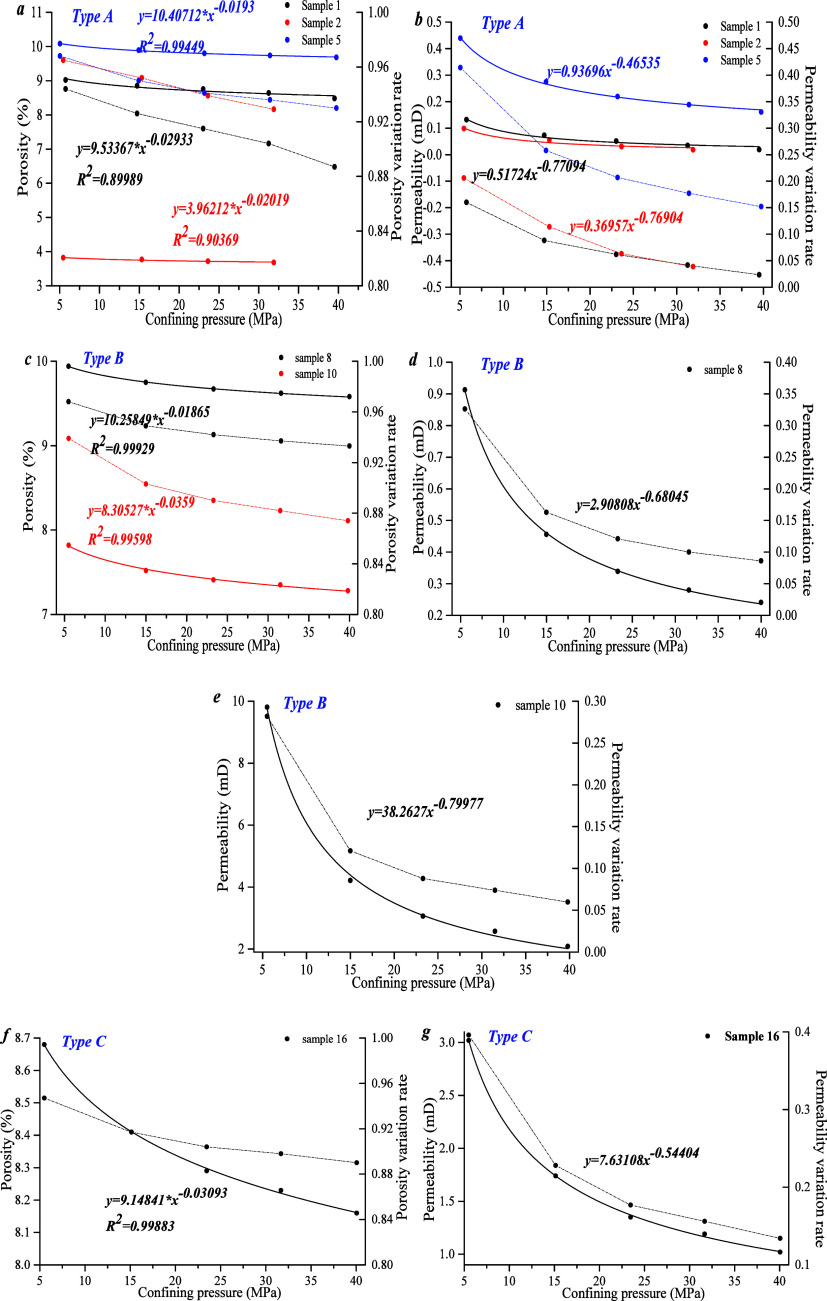
(a, b) Overpressure pore permeability of type A. (c–e) Overpressure pore permeability of type B. (f, g) Overpressure pore permeability of type C.

Figure 14a, c, and f show that initial porosity of type A ranges from 3.8 to 10.1%, and the initial porosity variation rate varies from 0.94 to 0.97. The initial porosity of type B is 7.8–10%, and the initial porosity variation rate is 0.94–0.97. The initial porosity of type C is 8.7%, and initial porosity variation rate is 0.95. The porosity and porosity variation rate of all samples decrease as a power function with increasing confining pressure. For a confining pressure of less than 15 MPa, the porosity and porosity variation rate of type B become larger; for a confining pressure above 15 MPa, changes in the porosity and porosity variation rate tend to be minimal. By comparison, the variation range of each sample in type B is small. The overall trend in porosity and porosity variation rate of type C is larger.

Similarly, the initial permeability of type A is 0.1–0.44 mD, and the initial permeability variation rate is 0.16–0.41; the initial permeability of type B is 0.91 mD, and the initial permeability variation rate is 0.33; the initial permeability of type C is 3.02 mD, and the initial permeability variation rate is 0.4 (Figure 14b,d,g). The permeability and permeability variation rates of all samples decrease as a power function with increasing confining pressure. For a confining pressure of less than 15 MPa, the permeability of type A varies greatly. For a confining pressure of more than 15 MPa, the variation of permeability tends to be gentle. However, the influence of confining pressure on the permeability variation rate can be divided into three stages. For a confining pressure of less than 15 MPa, the permeability variation rate is larger; for a confining pressure between 15 and 23 MPa, the permeability variation rate is slightly reduced; for a confining pressure greater than 23 MPa, the change range of the permeability variation rate tends to be gentle. In types B and C, the permeability and permeability variation rate are larger for a confining pressure less than 15 MPa. However, it is worth noting that the initial permeability of sample 10 is 9.8 mD, which is significantly higher than other samples, caused by microcracks produced during the sample preparation process (Figure 14e).

In conclusion, porosity, porosity change rate, permeability, and permeability change rate all decrease in a power function with increasing confining pressure. Moreover, it changes in stages with the confining pressure. When the confining pressure is less than 15 MPa, the changes in porosity and permeability are significant. When it is greater than 15 MPa, the changes decrease. This is because confining pressure can cause microscopic deformation between rock particles, resulting in a tighter arrangement of the particles. This type of intergranular compaction significantly reduces the porosity. Porosity is one of the main factors affecting the permeability. As the porosity decreases, the permeability also decreases accordingly. This is because there is a positive correlation between the permeability and porosity. Permeability has a larger variation range and is more affected by confining pressure variations in comparison to porosity.

**Figure 15:**
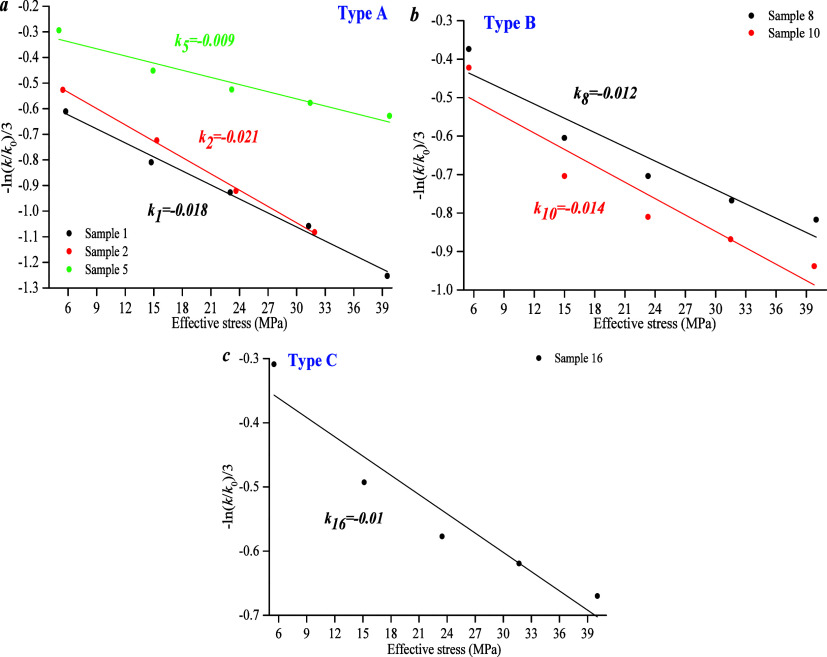
(a) Compression coefficient of type A. (b) Compression coefficient of type B. (c) Compression coefficient of type C.

Results show that there is a negative linear correlation between *p* and −ln(*k*/*k*0)/3. The compressibility coefficient of type A ranges from 0.018 to 0.021, that of type B ranges from 0.012 to 0.014, and that of type C is 0.01. Overall, the compressibility coefficient of type A is higher than that of types B and C (Figure 15), which could be influenced by the pore structure.

### 3.5 Pore-Permeability Dynamics Variation under the Effect of Pore and Fracture Structure Distribution Heterogeneity

**Figure 16:**
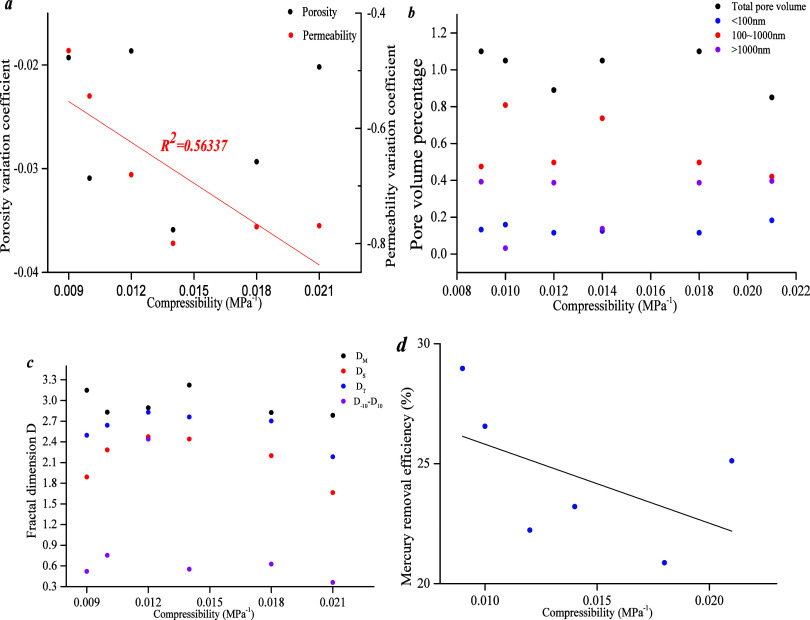
Relationship between compression coefficient and different values: (a) porosity and permeability variation coefficient; (b) pore volume percentage; (c) multifractal dimension; (d) mercury removal efficiency.

Figure 16a shows that compressibility has a weak negative linear correlation with the permeability variation coefficient and has no correlation with the porosity variation coefficient. Figure 16b,c shows that there is no significant correlation between compressibility and pore volume percentage. Figure 16d shows that there is a negative linear correlation between the compressibility coefficient and mercury removal efficiency. The mercury removal efficiency represents connectivity of pore fractures, indicating that the compressibility coefficient has a negative linear correlation with pore-fracture connectivity. However, compressibility has a stronger correlation with permeability.

**Figure 17:**
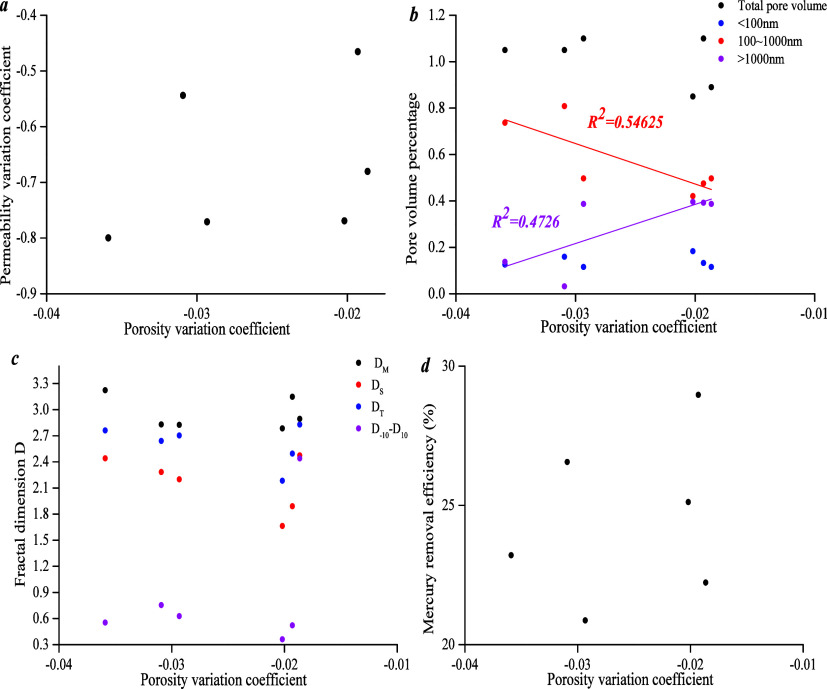
Relationship between porosity variation coefficient and different values: (a) permeability change coefficient; (b) pore volume percentage; (c) multifractal dimension; (d) mercury removal efficiency.

Figure 17b shows that the porosity variation coefficient has a weak negative linear correlation with meso-pore volume and a weak positive linear correlation with macro-pore volume, and it has no significant correlation with pore volume. It indicates that the porosity variation coefficient has a positive linear correlation with compressibility, and compressed space increases with higher porosity variation coefficient. Figure 17a,c,d shows that there is no clear correlation between the porosity variation coefficient and the different fractal dimensions, mercury removal efficiency, and permeability variation coefficient.

**Figure 18:**
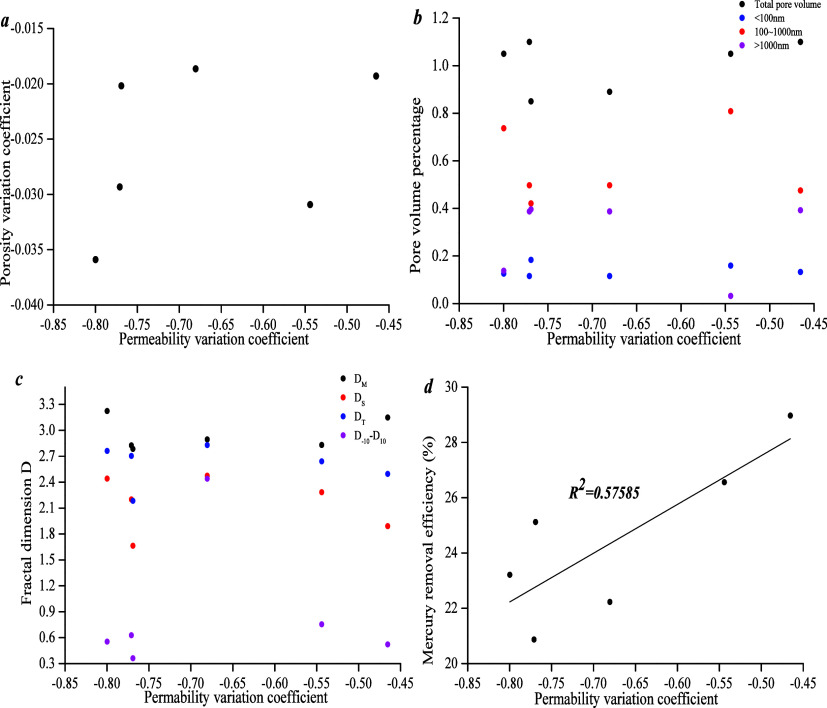
Relationship between permeability variation coefficient and different values: (a) porosity variation coefficient; (b) pore volume percentage; (c) multifractal dimension; (d) mercury removal efficiency.

Figure 18 shows that the permeability variation coefficient has a weak positive linear correlation with the mercury removal efficiency. There is no correlation with the porosity variation coefficient, different pore diameter volume percentage and fractal dimension. It is known that compressibility, porosity variation coefficient, and permeability variation coefficient have no clear correlation with pore structure parameters, which may be due to the fact that compressibility is affected by pore structure and mineralogical composition.

## 4 Conclusions

A total of 17 sandstone samples from the northwestern margin of the Junggar Basin in Xinjiang Province have been selected for the study of pore-fracture system distribution of target sandstones using SEM, HPMI, DP-P, and other testing techniques. Fractal calculations of target sandstone have been carried out applying the single- and multi-fractal theory. Effects of pore-fracture structure and distribution heterogeneity on porosity and permeability have been explored, the dynamic variations of production capacity under its constraints are clarified, and the following conclusions are obtained:

1. The M model represents the complexity of the rock surface area, whereas the S model represents the roughness of the rock pore volume. A comprehensive analysis shows that *D*_S_ has a significant relationship with pore volume percentage, and thus the S model could be more suitable for the quantitative characterization of the PFDH. The multifractal dimensions are consistent with those of the Sierpinski and Thermodynamic models, which indicates that there is consistency with single and multiple fractal models.
2. The porosity and permeability decrease as a power function with increasing confining pressure. There is a critical conversion pressure value as confining pressure increases. The pore permeability curves of different types of samples varied in stages, but the critical conversion pressure values were different. The permeability varies more in comparison to porosity and is more clearly affected by confining pressure. Pore compression space is affected by permeability and mercury removal efficiency.
3. The permeability variation coefficient has a positive linear correlation with mercury removal efficiency. There is no correlation with the porosity variation coefficient, different pore volume percentages, and fractal dimension. Compressibility, porosity variation coefficient, and permeability variation coefficient have no obvious relationship with pore structure parameters, which may due to the fact that compressibility of sandstone samples is affected by pore structure, mineral composition, and other factors.
